# Fabrication of a novel “all in one glove”- a functional tool for oral hygiene maintenance and the assessment of its effectiveness on plaque control in spastic cerebral palsy patients

**DOI:** 10.3389/froh.2024.1479684

**Published:** 2024-12-05

**Authors:** Sucharitha Palanisamy, Priyanka Cholan, Harinath Parthasarathy, Anupama Tadepalli

**Affiliations:** Department of Periodontics and Oral Implantology, SRM Dental College & Hospital, Chennai, India

**Keywords:** spasticity, cerebral palsy, toothbrush modifications, all in one, one single span, oral hygiene maintenance

## Abstract

**Background & objective:**

Spastic Cerebral Palsy (CP) is a neurological disorder from non-progressive brain injury or malformation during development, severely impairing movement, coordination, reflexes, posture, balance, and motor skills. Individuals with spastic CP experience limb spasticity, complicating tasks like precise gripping, particularly with toothbrushes. This study proposed a novel “all-in-one glove” for oral hygiene, assessing its efficacy and comparing it to conventional toothbrushes for spastic CP patients.

**Methods & materials:**

This randomized controlled trial was conducted on 30 clinically diagnosed spastic CP patients with Group 1 consisting of 15 Subjects with clinically diagnosed spastic CP with generalized gingival diseases using conventional oral hygiene techniques with weekly oral hygiene reinforcement and Group 2 consisting of 15 Subjects with clinically diagnosed spastic cerebral palsy with generalized gingival diseases using the novel “all in one glove” method for oral hygiene maintenance with weekly oral hygiene reinforcement. The Clinical Parameters assessed includes Full mouth plaque scores, Full mouth bleeding scores and OHI scores recorded at baseline, 3 and 6 months.

**Results:**

A statistically significant reduction was observed in Full-mouth Plaque, Bleeding, and Oral Hygiene Index (OHI) scores in both the groups (48.87% & 43.285%, 31.57% & 26.66%, 57.29% & 47.37% respectively). The Group 2 exhibited a marginally higher percentage reduction in all clinical parameters compared to the Group 1 at the measured time points.

**Conclusion:**

The utilization of the “All in one glove” has improved the effectiveness of oral hygiene maintenance and gingival health in spastic CP individuals.

**Clinical Trial Registration:**

[ClinicalTrials.gov], identifier, [REF/2022/06/055641].

## 1 Introduction

Cerebral palsy constitutes a neurodegenerative pathology distinguished by a multifaceted motor disorder arising from enduring motor cortex lesions (1). In the framework of the International Classification of Diseases (ICD) −10, cerebral palsy (CP) is denoted as G80 (2). Cerebral palsy is classified into different distinct categories based on the site of the injury to the cortex region amongst which spastic cerebral palsy is the predominant form, affecting approximately 80% of CP individuals. Impairments in spastic CP lead to increased muscle tone that leads to difficulties in voluntary movements, resulting in stiffness, awkward posture, and challenges in performing daily activities (3, 4).

Manifestations of spasticity in the upper limbs include flexion at the elbow, curvature of the wrist, and difficulties in coordinating grip strength (5, 6). Tasks demanding precise manual dexterity, encompassing activities such as grasping a toothbrush demands a delicate balance between the force exerted perpendicular to the interface surfaces, commonly referred to as tangential force and the grip-lift motion (7, 8). This imbalance between tangential load forces opposing the effects of gravity and the grip force in paretic hands exhibits distinctive features, including insufficient coordination between force derivatives compromising the efficiency of toothbrush manipulation capacity and maintaining hygiene in inaccessible areas leading to compromised oral wellness (9, 10). Besides spastic presentation in upper extremities, cerebral palsy patients commonly encounter difficulties in handling the complex mechanics associated with their oromotor function. These gestures may pose challenges in consistent rhythm of oral hygiene maintenance leading to multitude of oral health challenges which includes heightened prevalence of dental caries and periodontal disease (11, 12).

To address these challenges and to gear the motivation towards oral hygiene maintenance in spastic cerebral palsy patients, a novel “all-in-one glove” was conceptualized amalgamated with strategies such as patient education inclusive of both patients and caregivers and routine positive reinforcement. The present study assessed the effectiveness of the novel all in one glove in oral hygiene maintenance in spastic cerebral palsy patients and also to compare its efficacy with that of conventional toothbrushes routinely used by these patients.

## 2 Methods & materials

The current study is a Randomized Clinical Trial conducted in the Department of Periodontics, SRM Dental College, Ramapuram, Chennai. The research proposal was submitted to the institutional scientific and ethical review board and approval was obtained before the commencement of the study (SRMDC/IRB/2021/MDS/NO.502) and it was conducted adhering strictly to the guidelines by Helsinki Declaration of 1975. The product is granted with a published patent number on 9/2/2024 (PAN:202341071606).

### 2.1 Sample size calculation

The sample size determination was based on a study by Trupti Rai et al., calculated using G-Power version 3.1.9.2. With a power of 90%, an α error of 5%, and a substantial effect size, the required sample size was 15 per group, resulting in a total of 30 participants. Subjects were recruited from the outpatient clinic of the Department of Periodontics, SRM Dental College, Ramapuram, Chennai, and the Rehabilitation Clinic, Annanagar, Chennai, based on predefined inclusion and exclusion criteria. The study protocol was thoroughly explained to all participants and their parents/caretakers, and both verbal and written informed consent were obtained.

### 2.2 Procedure

The study commences in a sequential order which includes—3D design extraction, exportation of the 3D design into Flashforge Guider 2, choice of material, uploading of the material into the printer, fabrication of the glove, placement of the vibrators and battery connection and the essential oral hygiene components, allotment of gloves to the spastic CP patients, evaluation of the clinical parameters at three time points at baseline, 3 months and 6 months post non-surgical periodontal therapy (NSPT).

#### 2.2.1 3D design extraction

**Figure 1:**
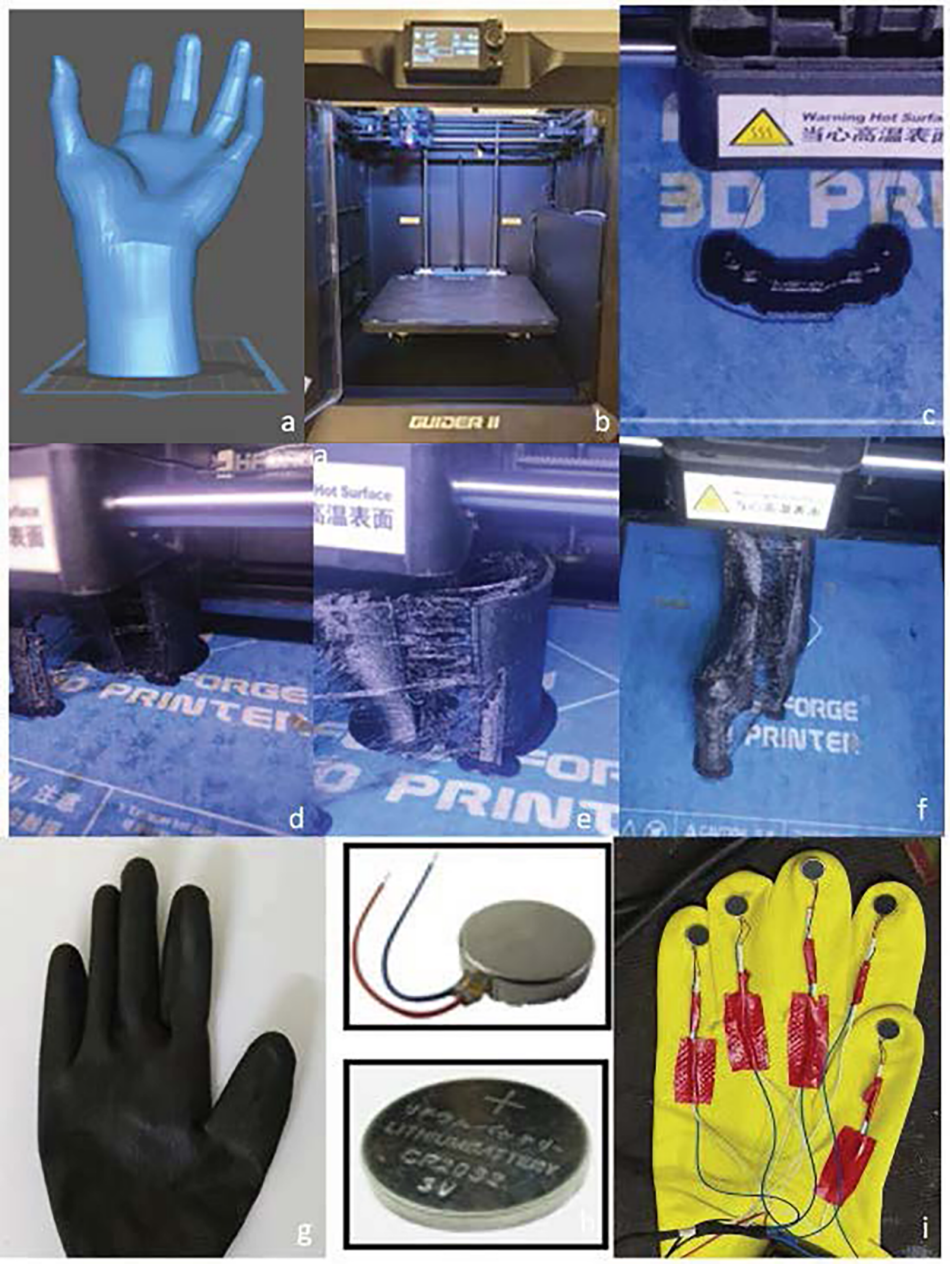
**(a)** 3D design of the glove. **(b)** Flashforge guider 2 3D printer. **(c)** Initial layout of the glove. **(d-f)** Layer by layer construction of the glove in both dorsal and ventral aspects. **(g)** Attachment of both the ventral and the dorsal aspect of the glove using polyglue. **(h)** The coin vibrators and the 3.7v lithium coin battery. **(i)** Alignment of the vibrators and the connecting wires soldered to the battery.

The 3D design is formulated using the 3D Modeling software (Autodesk Fusion 360) which are professional-grade CAD (Computer-Aided Design) programs used to create 3D models. Design considerations encompass adjustments in slit placement and size, enabling customization of the glove to smaller and medium sizes and supporting placement (Figure 1a).

#### 2.2.2 Exportation of the 3D design into the flashforge guider 2 printer

The obtained 3D design is exported in a format standard tessellation language (STL) and object file (OBJ). These formats contain the geometric codes needed for printing. The slicing software (PrusaSlicer) converts the 3D model into layers and generates the G-code. This G-code is transferred into a pen drive and is connected to the flashforge guider 2 printer which is calibrated appropriately.

#### 2.2.3 3D printing technology

The Flashforge Guider 3 employs vacuum-operated Fusion Deposition Modeling (FDM) technology (Figure 1b), utilizing filament materials to fabricate 3D models through a process of sequential layer deposition. The fundamental steps involved in the FDM technology includes filament feeding, extrusion and stratification of the material, layer by layer construction and solidification of the three dimensional object printed.

#### 2.2.4 Choice of material

The material of choice for the technology is polyurethane filament which is renowned for its remarkable adaptability and beneficial properties which includes biodegradability, positive biocompatibility, durability, flexibility, high resistance to wear and tear, and enhanced adaptability, making it a preferred material for our study.

#### 2.2.5 Fabrication of the glove

**Figure 2:**
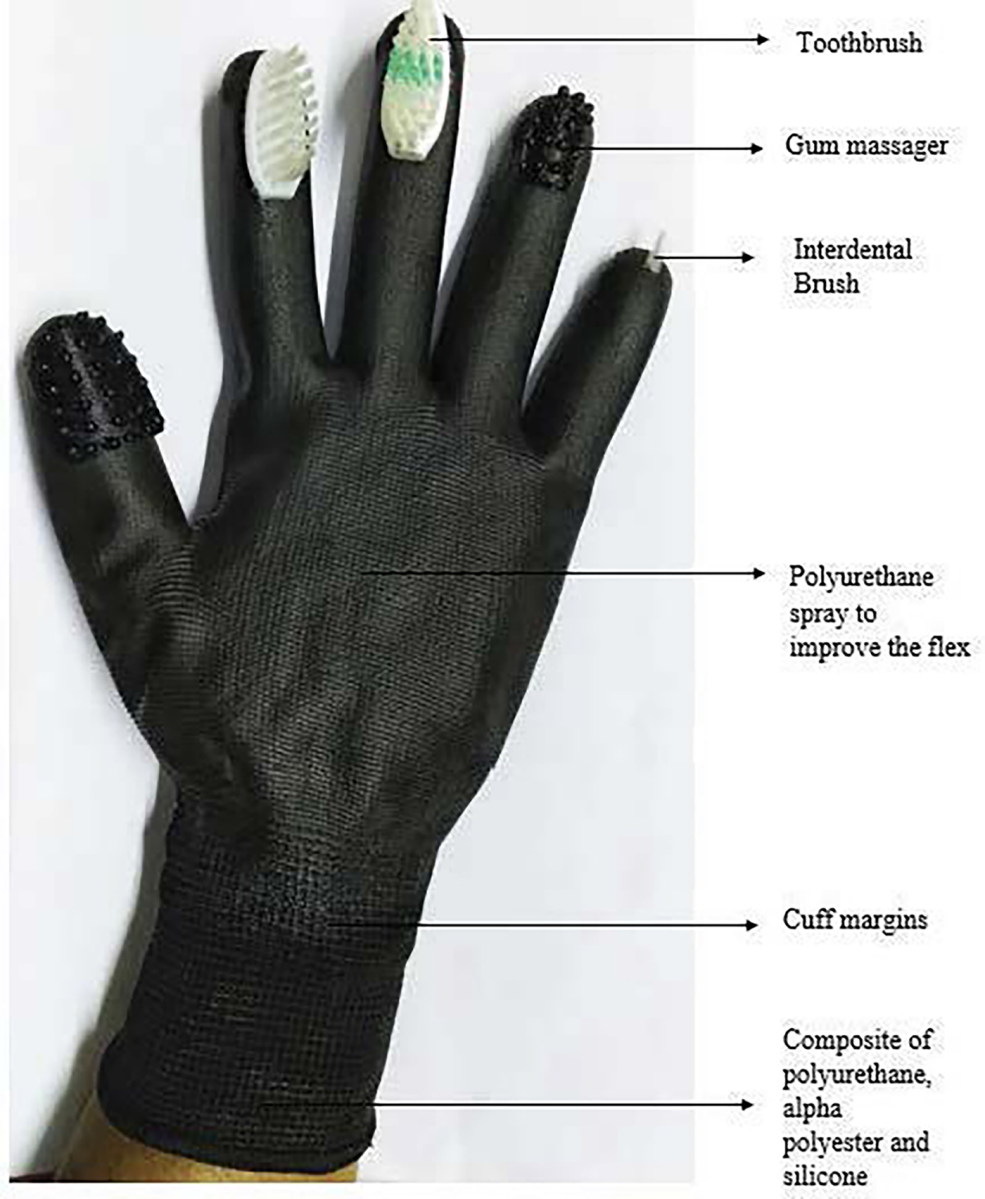
The novel “all in one glove”.

The polyurethane filament is loaded into the carriage and secured within the nozzle of the Flashforge Guider 2 printer. Preparing the printing platform involves heating it to 220°C, and the nozzle temperature is set to 150°C. The FDM printer initiates the additive manufacturing process by elevating the thermoplastic filament's temperature to its melting point. The extrusion nozzle systematically deposits the molten material in layers of 0.5 microns onto the build platform, adhering to the G-code instructions (Figure 1c). The build platform dynamically adjusts its position according to the design, facilitating the adhesion of each layer to the preceding one. This layer-by-layer methodology persists until the entire glove is meticulously constructed. The glove is printed into 2 segments which are dorsal and ventral aspects (Figures 1d–f). Post fabrication of the glove, the supports that provide additional support to the glove fabricated are removed (Figure 1g). To enhance wearability and ease of glove removal, the thickness at the cuff area was reduced and a composite material comprising 75% polyurethane, 15% polyester, and 10% silicon was utilized for the cuff area (Figure 2).

#### 2.2.6 Placement of the vibrators and battery connection and the essential oral hygiene components

This novel all in one glove employs a 3 V lithium cell coin battery placed within a battery holder which is soldered to the wires that connects it to the coin vibrators generating vibrations at approximately 12,000 Hz, akin to those produced by sonic electric toothbrushes (Figure 1h). To safeguard against external damage, both the battery holder and switch are soldered together and sealed within the cuff region of the glove. The oral hygiene tools are positioned as follows which includes the toothbrush head held between the index & middle fingers, while the gum massagers held between the thumbs and ring fingers (Figure 1i). The interdental aid is gripped by the little finger. Positioned behind the toothbrush head and gum massager components, the coin vibrator is incorporated.

#### 2.2.7 Allocation of the gloves in the spastic CP patients group

A sample of 30 patients are segregated into 2 groups which includes:

Group 1—subjects with clinically diagnosed spastic cerebral palsy with generalized gingival diseases using conventional oral hygiene techniques and tooth brushes with weekly oral hygiene reinforcement.

Group 2—subjects with clinically diagnosed spastic cerebral palsy with generalized gingival diseases using novel “All in one glove” for oral hygiene with weekly oral hygiene reinforcement.

#### 2.2.8 Evaluation of the clinical parameters

The clinical parameters encompass the use of the Full Mouth Plaque Index (Dr. Timothy J. O'Leary in 1972), Full Mouth Bleeding Index (Ainamo & Bay in 1975) and Simplified Oral Hygiene Index (John C Green and Jack R Vermillion in 1960) at baseline, 3 months, and 6 months’ recall intervals following NSPT.

## 3 Statistical analyses

Data analysis was conducted using SPSS (IBM SPSS Statistics for Windows, Version 23.0). Independent sample *t*-tests compared mean values between groups, while paired *t*-tests evaluated pre- and post-treatment comparisons. Chi-square tests assessed qualitative variable differences. Pearson correlation analysis examined clinical parameters at baseline, 3 months, and 6 months post-NSPT for both groups. Significance was set at *p* < 0.05 for all two-sided tests.

## 4 Results

Intragroup analysis amongst all the three clinical parameters inclusive of full mouth plaque, full mouth bleeding and simplified oral hygiene scores at baseline, 3 months, and 6 months for both Group 1 and Group 2 revealed a highly statistically significant difference (*p* < 0.001). Additionally, the intragroup analysis at 3 and 6 months for both groups demonstrated a statistically significant difference (Table 1). The intergroup comparison between Group 1 and Group 2 at the 3 and 6-month time points for the full mouth plaque index revealed highly statistically significant differences (*p* < 0.001). For the full mouth bleeding index and the simplified oral hygiene index, the comparative analysis indicated statistically significant differences (*p* < 0.05) (Table 2). The percentage reduction in the full mouth plaque index for Group 1 at the time intervals baseline to 3 months, baseline to 6 months, and 3 months to 6 months was 43.285%, 45.8571%, and 4.534%, respectively. For Group 2, the reductions were 48.87%, 51.2676%, and 4.683%, respectively. For the full mouth bleeding index, the percentage reduction in Group 1 at the time intervals baseline to 3 months, baseline to 6 months, and 3 months to 6 months was 26.66%, 30.6667%, and 5.455%, respectively. In Group 2, the reductions were 31.57%, 35.6725%, and 5.982%, respectively. Atlast, the percentage reduction in the simplified oral hygiene index for Group 1 at the time intervals baseline to 3 months, baseline to 6 months, and 3 months to 6 months was 47.37%, 55.3824%, and 15.208%, respectively. For Group 2, the reductions were 57.29%, 66.177%, and 20.8%, respectively (Table 3).

**Table 1:** Intragroup analysis and paired *t*-test values among group 1 and 2 at different time points—baseline, three months and six months.

Variable	Pairwise comparison		Group 1 *P*-value (Sig.)	Group 2 *P*-value (Sig.)
Full mouth plaque index (FMPI)	Baseline	3 months	*P* < 0.0001**	*P* < 0.0001**
	Baseline	6 months	*P* < 0.0001**	*P* < 0.0001**
	3 months	6 months	0.0164*	0.0395*
	Paired *t*-test (Bas Vs. 3 mon)		41.2523	34.7573
	Paired *t*-test (Bas Vs. 6 mon)		28.087	33.1595
Full mouth bleeding index (FMBI)	Baseline	3 months	*P* < 0.0001**	*P* < 0.0001**
	Baseline	6 months	*P* < 0.0001**	*P* < 0.0001**
	3 months	6 months	0.049*	0.0344*
	Paired *t*-test (Bas Vs. 3 mon)		11.129	16.099
	Paired *t*-test (Bas Vs. 6 mon)		12.911	17.244
Simplified oral hygiene (OHI-S)	Baseline	3 months	*P* < 0.0001**	*P* < 0.0001**
	Baseline	6 months	*P* < 0.0001**	*P* < 0.0001**
	3 months	6 months	0.05*	0.0009*
	Paired *t*-test (Bas Vs. 3 mon)		15.261	25.0614
	Paired *t*-test (Bas Vs. 6 mon)		18.5151	32.9667

*p < 0.05, statistically significant.

**p < 0.001, highly significant.

**Table 2:** Descriptive statistics pertaining to the clinical variables in groups 1 and 2 at baseline, three months and six months.

Variable	Time points	MEAN ± S.D		*T*-test	*P*-value (sig.)
		Group 1	Group 2		
Full mouth plaque index (FMPI)	Baseline	0.70 ± 0.025	0.71 ± 0.021	1.1862	0.245
	3 months	0.397 ± 0.013	0.363 ± 0.02	5.5204	0.0001**
	6 months	0.379 ± 0.024	0.346 ± 0.026	3.845	0.0006**
Full mouth bleeding index (FMBI)	Baseline	0.525 ± 0.033	0.513 ± 0.024	1.139	0.264
	3 months	0.385 ± 0.030	0.351 ± 0.025	3.372	0.002*
	6 months	0.364 ± 0.028	0.330 ± 0.0267	3.404	0.0021*
Simplified oral hygiene (OHI-S)	Baseline	2.824 ± 0.176	2.927 ± 0.205	1.4764	0.151
	3 months	1.486 ± 0.319	1.25 ± 0.214	2.3795	0.025*
	6 months	1.26 ± 0.304	0.990 ± 0.165	3.023	0.0053*

*p < 0.05, statistically significant.

**p < 0.001, highly significant.

**Table 3:** Mean percentage reduction in clinical variables in groups 1 and 2 at timepoints—baseline, three months, and six months post- NSPT.

Variables	Time points	Group 1	Group 2
Full mouth plaque index (FMPI)	% reduction (baseline—3 months)	43.285%	48.87%
	% reduction (baseline—6 months)	45.8571%	51.2676%
	% reduction (3 months–6 months)	4.534%	4.683%
Full mouth bleeding index (FMBI)	% reduction (baseline—3 months)	26.66%	31.57%
	% reduction (baseline—6 months)	30.6667%	35.6725%
	% reduction (3 months–6 months)	5.455%	5.982%
Simplified oral hygiene (OHI-S)	% reduction (baseline—3 months)	47.37%	57.29%
	% reduction (baseline—6 months)	55.3824%	66.177%
	% reduction (3 months–6 months)	15.208%	20.8%

## 5 Discussion

Spastic cerebral palsy (CP) is identified as a neurodegenerative condition with a complex motor disorder stemming from enduring lesions in the motor cortex. These disruptions during development affect milestones, muscle tonus, primitive reflexes, and grip force, creating challenges for individuals in holding and using a toothbrush effectively (13). This difficulty in precision grip and muscle coordination hampers their ability to hold the toothbrush and perform optimal oral hygiene, thereby elevating the risk of developing caries, gingival, and periodontal diseases (14, 15).

Individuals with spastic CP adhere to standard oral hygiene practices with basic toothbrushes, facing limited specialized design options compared to healthy counterparts (16). The newer adaptations primarily focus on the toothbrush handle or the adoption of electric toothbrushes, lacking a comprehensive approach covering all oral hygiene components (17). The absence of these adaptations results in an estimated plaque removal efficacy of approximately 62% through toothbrush use, leaving 30%–40% of plaque unaddressed (18). Modifications in toothbrush handle design often include features like an elongated shank, thick grips, flexible shanks, attaching handles to Velcro straps, or adding a tennis ball for improved grip (19–21). While these modifications enhance toothbrush handling, the improvements in oral hygiene status and interdental plaque control remain limited. This unaddressed plaque contributes to heightened inflammatory load, particularly in individuals with spastic cerebral palsy, where spasticity negatively impacts muscle tone and the effective force exerted by the paretic hand, leading to the gradual onset of gingival and periodontal diseases (22–24).

To address these drawbacks, the novel “all in one glove” aims to ease handling, overcoming difficulties associated with gripping a toothbrush. Additionally, the glove allows better access to inaccessible areas compared to traditional toothbrushes and integrates all oral hygiene components into a single unit. This all-in-one glove features toothbrushes on the index and middle finger pad, gum massagers and interdental aid on the ring and thumb finger pad, and little finger, respectively. To enhance effectiveness, the glove includes vibrators producing 12,000 Hz vibrations, similar to sonic toothbrushes, addressing challenges related to suboptimal load force during oral hygiene.

The significant decrease in plaque scores can be attributed to the glove's overall design with vibrators placed behind the toothbrushes, simulating a sonic toothbrush effect that disrupts accumulated plaque and calculus (Table 1). This difference may also be linked to increased glove usage frequency in the Group 2 and the motivational support provided through weekly teleconferencing sessions in both online and offline modes for both Group 1 & 2. This finding aligns with Helene Hoye et al.'s 2020 study, underscoring the substantial role of teleconferencing in guiding cerebral palsy individuals on their HRQoL and OHRQoL. Teleconferencing emphasizes temporal aspects, frequency, and self-driven motivation for engaging in oral hygiene practices, with the duration and frequency tailored to individual performance and dedication. Directed teleconferencing not only reinforces adopted methods but also provides training for consistent execution of oral care routines (25).

The decrease in bleeding scores is likely linked to the decrease in plaque and calculus accumulation. The reduction in plaque and calculus, coupled with the use of gum massagers, may have contributed to improving gingival health and minimizing the likelihood of gingival bleeding (Table 2). This aligns with a 2021 study by Nishu Singla et al., where oil gum massage therapy with gum massagers resulted in a significant decrease in plaque and gingival index scores (*P* < 0.05) (26). Similarly, a 1991 study by Bratel et al. noted a substantial reduction in plaque and bleeding scores (*P* < 0.005), attributed to regular recall visits and a well-defined oral prophylactic program (27).

The noteworthy decrease in OHI-S scores is credited to the vital role of vibrators in disrupting calculus and reducing plaque calculus load, enhancing the overall effectiveness of the all-in-one glove (Table 1). These vibrators, powered by a 3.7v lithium battery, ensure a consistent power supply for sustained efficacy. The reduction in OHI-S scores can also be attributed to the holistic approach of integrating all three oral hygiene components into a single entity. A 2021 study by Magda and colleagues emphasized the significance of sonic toothbrushes in oral hygiene maintenance, highlighting substantial differences in Plaque Index (PI), Gingival Index (GI), and Bleeding on Probing (BOP) compared to manual toothbrushing methods (28).

Although the all-in-one glove exhibited enhancements in full mouth plaque scores, full mouth bleeding scores, and OHI-S scores, it requires focused attention and modification in certain aspects. Limited color options in polyurethane material raise concerns, especially considering the preference for diverse colors in young adults with cerebral palsy. Future studies will address these considerations. The absence of storage covers for the glove was noted during the study and will be rectified in future research with the introduction of a utility box. Shelf life assessment of toothbrush and interdental aid components is crucial, considering the potential deterioration with inappropriate force application, as indicated in a study by Ni Zhou et al. (29). Minor modifications, such as removable dental hygiene components, can enhance usability. The current glove sizes are limited to small and medium, suggesting a need for customization based on factors like hand size and finger functionality.

This study marks the first exploration of the fabrication and use of the innovative all-in-one glove, presenting a holistic approach to oral hygiene for individuals with spastic cerebral palsy. Crafted from durable polyurethane with a four-year shelf life, the glove's practicality can be extended by introducing replaceable toothbrushes and interdental aids every two months. The elastic cuff, made of a polyurethane, polyester, and silicone composite, facilitates easy donning and doffing, enhancing usability for spastic CP patients. Additionally, the thermoplastic polyurethane material is environmentally friendly and economically feasible compared to electric toothbrushes. The glove's comprehensive approach demonstrated substantial mechanical plaque control in individuals with spastic cerebral palsy. Future research should consider larger sample sizes and diverse cohorts for a deeper understanding of the glove's functionality and material properties, potentially broadening its usability across age groups and manual dexterity levels.

## 6 Conclusion

The novel “all in one glove” would augment the handling efficiency and foster patient motivation towards oral hygiene maintenance, consequently yielding favorable outcomes in OHRQoL and gingival health among individuals with spastic cerebral palsy.

## Data Availability

The original contributions presented in the study are included in the article/Supplementary Material, further inquiries can be directed to the corresponding author.

## References

[1] Bax M Goldstein M Rosenbaum P Leviton A Paneth N Dan B Proposed definition and classification of cerebral palsy, April 2005. Dev Med Child Neurol. (2005) 47(8):571–6. 10.1017/S001216220500112X 16108461

[2] World Health Organization, Others. International statistical Classification of diseases and related health problems. 10th Revision, Vol. 2. Geneva: World Health Organization (2022).

[3] Rosenbaum P. What causes cerebral palsy? Br Med J. (2014) 349:g4514. 10.1136/bmj.g4514 25026893

[4] Hugos CL Cameron MH. Assessment and measurement of spasticity in MS: state of the evidence. Curr Neurol Neurosci Rep. (2019) 19:79. 10.1007/s11910-019-0991-2 31471769 PMC6948104

[5] Nicholson JH Morton RE Attfield S Rennie D. Assessment of upper-limb function and movement in children with cerebral palsy wearing lycra garments. Dev Med Child Neurol. (2001) 43(6):384–91. 10.1017/s001216220100072x 11409827

[6] Bass N. Cerebral palsy and neurodegenerative disease. Curr Opin Pediatr. (1999) 11:504–7. 10.1097/00008480-199912000-00005 10590907

[7] Rapp CE Jr Torres MM. The adult with cerebral palsy. Arch Fam Med. (2000) 9:466–72. 10.1001/archfami.9.5.466 10810953

[8] Mato EG López LS Freitas MD Pazos MTA Posse JL Dios PD Plaque removal efficacy of a new toothbrush with a double-sided head and rotating handle-a pilot randomized control trial in acquired brain injury patients. Clin Oral Investig. (2023) 27(8):4855–60. 10.1007/s00784-023-05106-y 37389693 PMC10415501

[9] Butler C Campbell S. Evidence of the effects of intrathecal baclofen for spastic and dystonic cerebral palsy. Dev Med Child Neurol. (2000) 42:634–45. 10.1111/j.1469-8749.2000.tb00371.x 11034458

[10] Strauss D Cable W Shavelle R. Causes of excess mortality in cerebral palsy. Dev Med Child Neurol. (1999) 41:580–5. 10.1111/j.1469-8749.1999.tb00660.x 10503915

[11] Chandra Pani S AlEidan SF AlMutairi RN AlAbsi AA Nasser AlMuhaidib D Faisal AlSulaiman H The impact of gross motor function on the oral health-related quality of life in young adults with cerebral palsy in Saudi Arabia. Int J Dent. (2020) 2020:4590509. 10.1155/2020/4590509 32190052 PMC7064833

[12] Bensi C Costacurta M Docimo R. Oral health in children with cerebral palsy: a systematic review and meta-analysis. Spec Care Dentist. (2020) 40:401–11. 10.1111/scd.12506 32815638

[13] Daraniyagala TR Herath CK Gunasinghe MS Ranasinghe N Herath MB Jayasooriya PR. Oral health status of children with cerebral palsy and its relationship with caregivers' knowledge related to oral health. J South Asian Assoc Pediatr Dent. (2019) 2(2):37–42. 10.5005/jp-journals-10077-3031

[14] Afifah H Prijatmoko D Kiswaluyo K. Effectiveness wall mounted automatic toothbrush against oral hygiene on cerebral palsy children at SMPLB and SMALB D YPAC Jember. J Dentomaxillofac Sci. (2020) 4:180–3. 10.15562/jdmfs.v4i3.956

[15] Sedky NA. Assessment of oral and dental health status in children with cerebral palsy: an exploratory study. Int J Health Sci. (2018) 12:4–14. PMC5870305 29623011

[16] Soncini JA Tsamtsouris A. Individually modified toothbrushes and improvement of oral hygiene and gingival health in cerebral palsy children. J Pedod. (1989) 13:331–4. 2534698

[17] Ferraz NK Tataounoff J Nogueira LC Ramos-Jorge J Ramos-Jorge ML Pinheiro ML. Mechanical control of biofilm in children with cerebral palsy: a randomized clinical trial. Int J Paediatr Dent. (2015) 25(3):213–20. 10.1111/ipd.12132 25200983

[18] Yitzhak M Sarnat H Rakocz M Yaish Y Ashkenazi M. The effect of toothbrush design on the ability of nurses to brush the teeth of institutionalized cerebral palsy patients. Spec Care Dentist. (2013) 33(1):20–7. 10.1111/j.1754-4505.2012.00311.x 23278145

[19] Puthiyapurayil J Anupam Kumar TV Syriac G R M KT R Najmunnisa. Parental perception of oral health related quality of life and barriers to access dental care among children with intellectual needs in Kottayam, central Kerala-A cross sectional study. Spec Care Dentist. (2022) 42(2):177–86. 10.1111/scd.12658 34614254

[20] Pasiga BD. Utilization of special grip toothbrushes for children with cerebral palsy. Sys Rev Pharm. (2020) 11(8):9–16.

[21] Rai T YM K Rao A P AN Natarajan S Joseph RM. Evaluation of the effectiveness of a custom-made toothbrush in maintaining oral hygiene and gingival health in cerebral palsy patients. Spec Care Dentist. (2018) 38(6):367–72. 10.1111/scd.12334 30350870

[22] Yoshida RA Gorjão R Mayer MPA Corazza PFL Guare RO Ferreira ACFM Inflammatory markers in the saliva of cerebral palsy individuals with gingivitis after periodontal treatment. Braz Oral Res. (2019) 33:e033. 10.1590/1807-3107bor-2019.vol33.0033 31269113

[23] de Gutierrez GM Marin LM Xiao Y Escalante-Herrera A Santos MTBR Siqueira WL. Detection of periodontal disease activity based on histatin degradation in individuals with cerebral palsy. Heliyon. (2022) 8(8):e10134. 10.1016/j.heliyon.2022.e10134 36046535 PMC9421316

[24] Chu CH Lo ECM. Oral health status of Chinese teenagers with cerebral palsy. Community Dent Health. (2010) 27:222–6. 21473357

[25] Høye H Jahnsen RB Løvstad M Hartveit JF Sørli H Tornås S A mindfulness-based stress reduction program via group video conferencing for adults with cerebral palsy - a pilot study. Front Neurol. (2020) 11:195. 10.3389/fneur.2020.00195 32318010 PMC7146892

[26] Singla N Acharya S Martena S Singla R. Effect of oil gum massage therapy on common pathogenic oral microorganisms - a randomized controlled trial. J Indian Soc Periodontol. (2014) 18(4):441–6. 10.4103/0972-124X.138681 25210256 PMC4158583

[27] Bratel J Berggren U. Long-term oral effects of manual or electric toothbrushes used by mentally handicapped adults. Clin Prev Dent. (1991) 13:5–7. 1832101

[28] Mensi M Scotti E Sordillo A Brognoli V Dominici MP Calza S. Efficacy of sonic versus manual toothbrushing after professional mechanical plaque removal: a 6-month randomized clinical trial. Int J Dent Hyg. (2021) 19(4):366–75. 10.1111/idh.12541 34328264 PMC9292217

[29] Zhou N Wong HM McGrath C. Toothbrush deterioration and parents’ suggestions to improve the design of toothbrushes used by children with special care needs. BMC Pediatr. (2020) 20:443. 10.1186/s12887-020-02347-8 32958022 PMC7504597

